# 4Gbaud PS-16QAM D-Band Fiber-Wireless Transmission over 4.6 km by Using Balance Complex-Valued NN Equalizer with Random Oversampling

**DOI:** 10.3390/s23073655

**Published:** 2023-03-31

**Authors:** Tangyao Xie, Jianguo Yu

**Affiliations:** Beijing Key Laboratory of Work Safety Intelligent Monitoring, Beijing University of Posts and Telecommunications, Beijing 100876, China

**Keywords:** CVNN equalizer, D-band, PS-16QAM, balanced random oversampling

## Abstract

D-band (110–170 GHz) is a promising direction for the future of 6th generation mobile networks (6G) for high-speed mobile communication since it has a large available bandwidth, and it can provide a peak rate of hundreds of Gbit/s. Compared with the traditional electrical approach, photonics millimeter wave (mm-wave) generation in D-band is more practical and effectively overcomes the bottleneck of electrical devices. However, long-distance D-band wireless transmission is still limited by some key factors such as large absorption loss and nonlinear noises. Deep neural network algorithms are regarded as an important technique to model the nonlinear wireless behavior, among which the study on complex-value equalization is critical, especially in coherent detection systems. Moreover, probabilistic shaping is useful to improve the transmission capacity but also causes an imbalanced machine learning issue. In this paper, we propose a novel complex-valued neural network equalizer coupled with balanced random oversampling (ROS). Thanks to the adaptive deep learning method for probabilistic shaping-quadrature amplitude modulation (PS-QAM), we successfully realize a 135 GHz 4Gbaud PS-16QAM with a shaping entropy of 3.56 bit/symbol wireless transmission over 4.6 km. The bit error ratio (BER) of 4Gbaud PS-16QAM can be decreased to a soft-decision forward error correction (SD-FEC) with a 25% overhead of 2 × 10^−2^. Therefore, we can achieve a net rate of an 11.4 Gbit/s D-band radio-over-fiber (ROF) delivery over 4.6 km air free wireless distance.

## 1. Introduction

Numerous photonics-based methods for D-band (110–170 GHz) millimeter wave (MMW) signal generation demonstrated thus far include schemes using the optical frequency comb [1,2], optical heterodyne [3,4], etc. However, there are some drawbacks of the optical frequency comb method: (1) there is a high demand on the frequency of the radio frequency (RF) source in order to generate the broadband optical frequency comb. (2) A precise control of direct current (DC) bias and RF driving voltage on the external modulator is required to generate the flat optical frequency comb. (3) Additional devices are required such as a frequency multiplier and an optical filter. (4) The frequency of the generated mm-wave cannot be adjusted flexibly because it is an integer multiple of RF frequency. (5) Only two optical subcarriers of the frequency comb are selected to generate heterodyne mm-wave signals via a photodiode (PD), which results in power consumption. Therefore, the method using two independent free-run laser diodes is superior to that using the frequency comb, which does not suffer from the power consumption and high cost and performs better in terms of frequency adjustment flexibility. Yet there are several technical issues, i.e., the rapid attenuation of D-band in air space holds back the high-speed wireless transmission, which usually employs high-order QAM formats to further improve the spectrum efficiency [5,6], and it faces a fundamental signal-to-noise ratio (SNR) limitation, especially in the D-band long-distance transmission channel. Probabilistic shaping QAM (PS-QAM), as a step closer to the Shannon limit, brings an SNR gain of up to 1.53 dB compared with the regular QAM modulation [7]. Thus, it can relax the requirement of an SNR at the receiver side.

While for long-range high-capacity D-band PS-QAM wireless transmission is up to km and tens of Gbit/s scale, the major challenge is also found in the nonlinear noise. The main source of nonlinearity originates from four aspects as follows: (a) the large optical power from fiber; (b) the optoelectronic devices including modulators and PD; (c) the electrical amplifier (EA) and the high-power amplifier (HPA) in the wireless channel; and (d) the nonlinear impairments from mixers during down conversion. The constant modulus algorithm (CMA), known as a linear and decision-feedback equalizer, uses tap delay line filters to equalize a modulated signal and remove inter-symbol interference (ISI). However, its higher residual mean square error (MSE) is a major challenge for equalizing nonlinear channels in low SNR conditions. Deep neural network algorithms are regarded as an important technique to model the nonlinear wireless behavior, among which the study on complex-value equalization is critical, especially in coherent detection systems [8].

Although PS has become a useful approach to increase the capacity, the digital signal processing (DSP) process compatible with the shaped signal has become more challenging, especially in high-speed communication systems. Recently, we realized 2 Gbaud PS-16QAM D-band wireless transmission over 4.6 km by using Echo State Network-based Nonlinear Equalization. However, the proposed equalizer processes the real and imaginary signal, respectively, regardless of the phase information and DSP compatibility with PS [9]. Therefore, it is meaningful to investigate a single neural network processing both I and Q components together. Moreover, PS might hinder the performance obtained by the existing DSP algorithms. Traditional time-domain blind equalization algorithms, including CMA [10] and the Decision-Directed Least Mean Square (DD-LMS) algorithm [11], are usually affected by the uneven occurrence probability of constellation points when working on PS-QAM signals [12]. The truncated PS-64QAM modulation format is designed to obtain a more suitable shaping depth, and make the shaped signal better suited to the CMA equalization [13]. Actually, the recovery of received m-QAM signals can be regarded as an m-classification problem. Since the probability of m-QAM varies with the amplitude, there have been difficulties in learning the useful information from the minority class. Thus, lots of efforts are underway to handle this issue, i.e., oversampling, undersampling, etc. [14]. Among them, random oversampling (ROS) is very competitive due to its simplicity [15].

In this paper, we experimentally realize photonics-aided D-band m-QAM mm-wave transmission over a 4.6 km wireless distance. In order to increase the data rate, 4 Gbaud PS-16QAM transmission with a shaping entropy of 3.56 bit/symbol is achieved by deploying our proposed ROS in combination with a complex-valued neural network (CVNN) equalizer. The received PS-16QAM signal is firstly data processed via random oversampling and then nonlinear equalized by CVNN. The BER of 4Gbaud PS-16QAM can be decreased to SD-FEC with a 25% overhead of 2 × 10^−2^, and the data rate is 4Gbaud × 3.56 bit/symbol = 14.24 Gbit/s. Therefore, we can achieve a net rate of 4Gbaud × 3.56bit/symbol × (1−0.2) = 11.4 Gbit/s D-band ROF delivery over a 4.6 km air free wireless distance.

## 2. Operation Principle of Deep Learning Algorithms for PS-m-QAM Modulation

**Figure 1:**
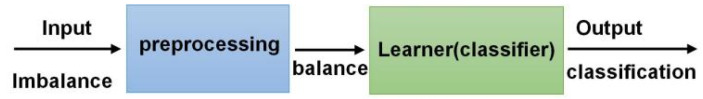
The approach for preprocessing before NN classifier.

In our conducted PS-m-QAM training scheme, the main digital signal processing (DSP) involves two steps in Figure 1: the preprocessing step aims to reduce the learning imbalance and QAM classification into *m* classes via a complex-valued neural network.

### 2.1. Data Preprocessing Method of Random Oversampling

**Figure 2:**
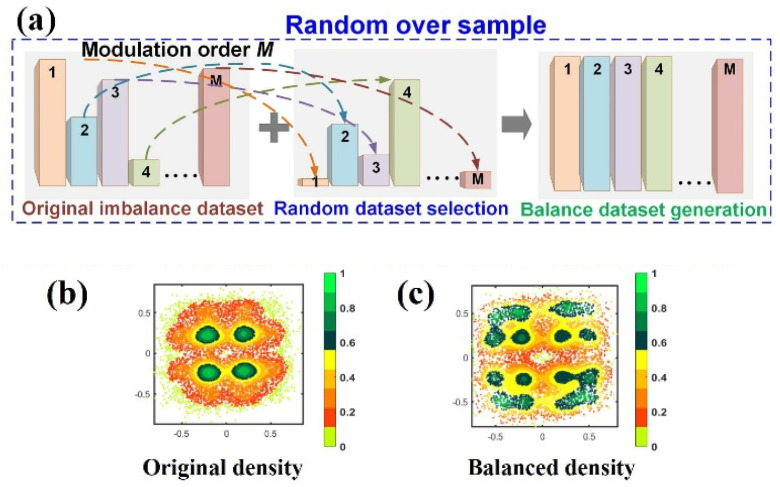
(**a**) Random oversampling principle for PS-m-QAM. The density of (**b**) original received PS-16QAM signals and (**c**) balance PS-16QAM signals. Color Bars: (Green: High Density, Orange Yellow: Regular Density, Bright Yellow: Sparse Density).

For the NN classification algorithm, class imbalance occurs when the number of samples in some classes is more than that of instances in other classes. Here, we define the larger dataset as the majority class, while the smaller one is called the minority class. An effective proposed method deals with the imbalance issue, allying a known ROS approach with a simple data cleaning method in Figure 2a. 

M-order QAM equalization can be considered as m-classification, while PS generates majority classes in inner rings and minority classes in outer rings. However, the initial PS m-QAM training set causes the NN model to be biased towards majority class problems, and it is difficult to learn with minority classes. It is called imbalance classification, in Figure 2a; the original imbalance dataset $T_{Original}$ for m-QAM symbols contains data with M different labels (C), and the number of samples $N_{C}$ between them is unbalanced. To solve the imbalance problem, ROS is an effective approach to ensure that different classes of the training set have the same number of samples. Based on the final required balance dataset $\left(T_{Balance}\right)$ size $N_{T}$, and according to the principle that the samples of M classes in $T_{Balance}$ is equal, the number of samples of each class can be calculated as $N_{B}=\frac{N_{T}}{M}$. To satisfy this condition, ROS randomly picks data from each class C in $T_{Original}$ to build $T_{Balanced}$. When M=16, it is the original density of PS-16-QAM trained data given in Figure 2b. The degree of class imbalance is increased with the larger shaping depth. Then the samples are randomly replicated according to the size of the training data and the inverse ratio between m classes. In general, a large size of the training data in minority classes is repeated, while a small subset of the majority class is randomly extracted. ROS enables the neural network to extract training samples of all classes with uniform probability in Figure 2c, bringing gains in the performance of equalizers. It is worth noting that although the training length is increased because of ROS, the capacity of the training sample set is unchanged. In other words, it implies that the additional epochs are selectively applied to the minority class rather than the majority class.

### 2.2. Deep Learning Complex-Value Network for m-QAM Data Input

**Figure 3:**
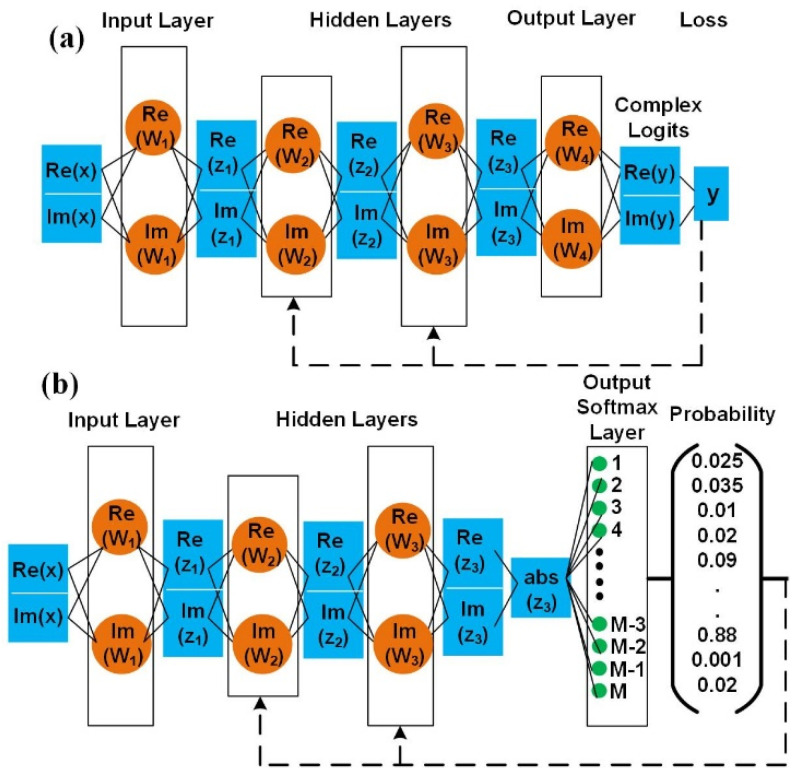
The schematic structure complex-valued NN (**a**) equalizer with MSE loss function, (**b**) classifier with a cross entropy loss function. Orange circles: neurons in the network, Blue squares: input and output data.

Generally, both I and Q parts are divided. The separated real and imaginary components of the complex m-QAM signal are trained via two real-valued NN classifiers, respectively. However, this problem is becoming increasingly important where the complex nature of the m-QAM signal cannot be ignored, especially its sensitivity to noises. To solve this problem, we present a complex-valued network. First, the complex training set X is fed into a fully connected CVNN as shown in Figure 3. The real-valued neural networks often use Re*LU* as the activation function, and the function Re*LU* can be expressed as:

(1) ReLU(x)=max(0,x)

We employ complex active function ℂRe*LU* [16] in Equation (2) to accurately model the nonlinearity of the complex channel.

(2) ℂReLU=ReLUreal+i·ReLUimaginary=max(0,real)+i·max(0,imaginary)

As we know, there are a multitude of common loss functions in a deep neural network such as mean square error (MSE) loss [17], cross-entropy (CE) loss [18], L1 loss [19], hinge loss [20], etc. One of which, the loss function deployed in the nonlinear equalization (NLE) framework, is the mean square error (MSE) loss and given as,

(3) $$e_{n}=Mean[{\left(T_{n}-O_{n}\right)}^{2}]$$

where $T_{n}\in {\mathbb{C}}^{N}$ is the target complex signal, and $O_{n}\in {\mathbb{C}}^{N}$ is the output complex value from the CVNN equalizer in Figure 3a.

In addition, CE has a superiority in the term of convergence speed and is often used as a reasonable loss function for classification tasks [21,22]. Actually, m-QAM equalization can also be regarded as multiclassification as in Figure 3b. For example, to solve the 64-QAM classification (CF) problem, the softmax function is a generalization of the logistic function that maps a length-T signal series of real values to a length-64 probability vector. In this manner, the sum of the output vector ${\left[p_{0}^{t},p_{1}^{t},p_{2}^{t},p_{3}^{t}\ldots p_{63}^{t}\right]}^{T}(t=1,2,\ldots P)$ equals 1, namely, $\sum \left(p_{0}^{t},p_{1}^{t},p_{2}^{t},p_{3}^{t}\ldots p_{63}^{t}\right)=1$. Specifically, the probability of the t-th symbol is given as below,

(4) $$p_{v}^{t}=soft\max(z_{v}^{t})=\frac{\exp(z_{v}^{t})}{\sum_{v\prime =0}^{63}\exp(z_{v\prime }^{t})}(v\in 0,1,2,3,...63)$$

where $z^{t}={\left[z_{0}^{t},z_{1}^{t},z_{2}^{t},z_{3}^{t}\ldots z_{63}^{t}\right]}^{T}$ is calculated as the absolute value of the complex signal from the last hidden layer, and is also the input vector into the output softmax layer at time *t*. In multiclassification applications, we often use cross entropy as the loss function in Equation (5),

(5) $$\begin{array}{cc} \begin{array}{l} Loss=-\sum_{v}s_{v}^{t}\ln p_{v}^{t}\\ =-s_{u}^{t}\ln p_{u}^{t}=-\ln p_{u}^{t} \end{array} & \left(\left\{\begin{array}{l} s_{v}^{t}=0(v\neq u)\\ s_{v}^{t}=1(v=u) \end{array}\right.\right) \end{array}$$

where $s_{v}^{t}$ is illustrated as the target 64-QAM signal at time t, and Equation (4) substituted into the derivate function of Equation (5) is calculated as,

(6) $$\frac{\partial Loss}{\partial u}=\frac{\partial \ln p_{u}^{t}}{\partial u}=\frac{\partial (-\ln\frac{\exp(z_{u}^{t})}{\sum_{v\prime =0}^{63}\exp(z_{v\prime }^{t})})}{\partial u}=p_{u}^{t}-1$$

The given result from Equation (6) is the gradient updating according to the backpropagation algorithm, so that the connected weight vector can be iteratively updated until the desired epoch or error value is reached. 

In terms of complexity, MSE is superior to CE because there is only one neuron unit in the output layer of CVNN NLE, while there are M units in the output softmax layer of CVNN CE. In terms of precision, on the one hand, CE loss weight updates faster than MSE. On the other hand, MSE is a nonconvex optimization, while cross entropy is a convex one. Thus, CE avoids falling into a local optimal solution and is more convenient, especially for the multiclassification optimization. Thus, we should consider the two aspects of complexity and training precision to select the optimal loss function.

Regarding the technical details, ROS randomly replicates the data within each minority class until all classes are balanced. We use the stochastic gradient descent (SGD) algorithm in the training of the neural network. During the training process, the training status depends on the average loss of the network model in the verification set. The optimal model in the structure can be obtained until the average loss no longer decreases.

ROS enables SGD to extract training samples of all classes from the training set with uniform probability to obtain performance improvement, otherwise, the imbalance distribution of the initial dataset may cause the model to be biased towards the majority class.

## 3. Experimental Setup

**Figure 4:**
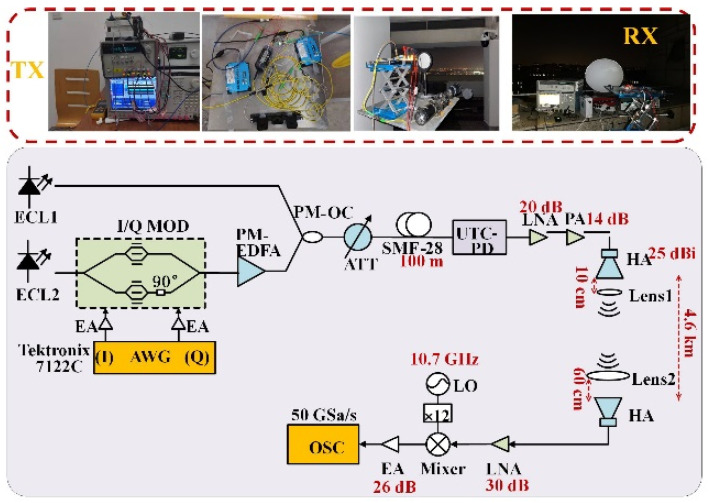
Experiment setup and photos of 135 GHz fiber-wireless system over 4.6 km free-space distance.

The experimental setup of illustrated photonics-aided mm-wave communication through 4.6 km free-space link is presented in Figure 4. m-QAM modulation formats such as PS-16QAM generated by AWG (Tektronix 7122C) are used to drive I and Q branch of the I/Q modulator (FTM7961EX, 32 GHz bandwidth, with a 3 dB bandwidth of 30 GHz, manufacturer: Fujitsu, Japan). It is worth noting that the probabilistic amplitude shaping (PAS) scheme is employed for I and Q branch, respectively. Constant composition distribution matcher (CCDM) is combined with DVB-S2 LDPC, which supports bit-interleaved coded modulation (BICM). The information entropy of the transmitted PS-16QAM signal is adjusted as 3.56 bit/symbol. The optical signal from ECL 2 is modulated by I/Q modulator and amplified by a polarization-maintaining erbium-doped fiber amplifier (PM-EDFA, Amonics AEDFA-PM-23-B-FA, manufacturer: Amonics, Hong Kong), then combined with the other optical signal generated by ECL1 by a polarization-maintaining coupler (PM-OC), the frequency space between them is 135 GHz, and the combined optical beam is optimized by an attenuator (ATT) and passes through a 100 m SMF-28 and finally beats in a Uni-Traveling-Carrier Photodiode (UTC-PD) and generates a sub-THz (~135 GHz, D-band) signal. After the two-stage amplification (LNA with 20 dB gain and PA with 14 dB gain), the D-band mm-wave signal is transmitted from the Horn antenna (HA), and travels over 4.6 km in the free-space link. Here, HA has a gain of 25 dBi. 

In addition, Lens 1 and Lens 2 are placed at the receiving end and the transmitting end, respectively. Lens 1 has a focal length of 10 cm and Lens 2 has a focal length of 60 cm. At the receiving end, after the signal is detected by the HA identical to the one at transmit side, it is first amplified by a low noise amplifier (LNA) with a gain of 30 dB, then mixed with a 131.4 GHz (10.95 × 12 = 131.4 GHz) signal in a mixer, generates a 3.6 GHz (135−10.95 × 12 = 3.6 GHz) IF signal, and is amplified by an EA with a gain of 26 dB and finally captured by 50 GSa/s OSC. The offline DSP at Rx is followed by down conversion, resampling, CMA, frequency offset estimation (FOE), carrier phase recovery (CPR), Gram–Schmidt orthogonalization process (GSOP), DD-LMS, and deep neural training.

## 4. Experimental Results

**Figure 5:**
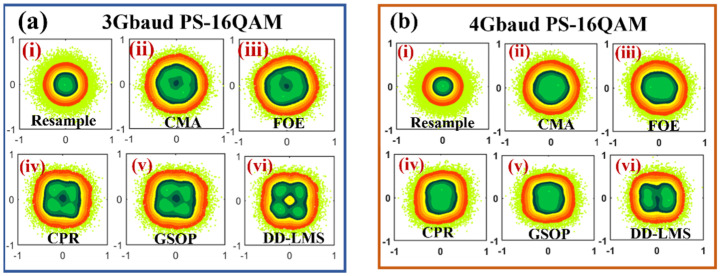
The density distribution of constellation diagrams for (**a**) 3Gbaud PS-16QAM and (**b**) 4Gbaud PS-16QAM, respectively, after (i) resample; (ii) CMA; (iii) FOE; (iv) CPR; (v) GSOP, and (vi) DD-LMS. Green: High Density, Orange Yellow: Regular Density, Bright Yellow: Sparse Density.

We give the corresponding density distribution of the constellation diagrams after resample, CMA, FOE, CPR, GSOP, and DD-LMS as Insets (i)–(vi) in Figure 5. We can see that the constellation diagram of the 3Gbaud PS-16QAM signal is improved with the above traditional DSP algorithms. However, the constellation diagrams of signals remain fuzzy because these steps are linear and useless for nonlinear noises. It can be seen from Inset (vi) in Figure 5b that the four constellation points of the 4Gbaud PS-16QAM signal cannot even be identified in the inner circle. It implies that the PS-16QAM signals with a higher baud rate are more easily affected by noises and have a higher SNR requirement. In order to conquer the nonlinear problem, various nonlinear training algorithms are deployed.

**Figure 6:**
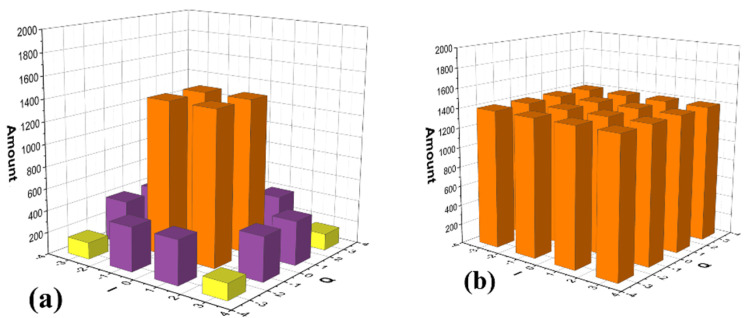
Transmitted PS-16QAM constellation points distribution (**a**) before ROS, (**b**) after ROS. Bars: (Orange: Large amount, Purple: Medium amount, Yellow: Small amount.)

As shown in Figure 6a, the transmitted PS-16QAM has an uneven distribution changing with the information entropy ν. Before ROS, most of the transmitted PS-16QAM signals in the inner circle are generated, while the outer circles contribute to the minority, and it is especially serious with a smaller ν. In order to overcome the imbalance learning problem, we propose a CVNN nonlinear classifier algorithm combined with a balancing ROS method. 

First, the unbalanced PS-16QAM training data are inverse oversampled from the original dataset. It should be noted that ROS is only used for training sequences, while the original test data are still used to validate the proposed neural structure’s validity. In our experiment, ν is set as 3.56 bit/symbol. After ROS processing, the target output is 26,864 when the training size is 11,000. Thus, the corresponding target output has an equal amount distribution in Figure 6b.

**Figure 7:**
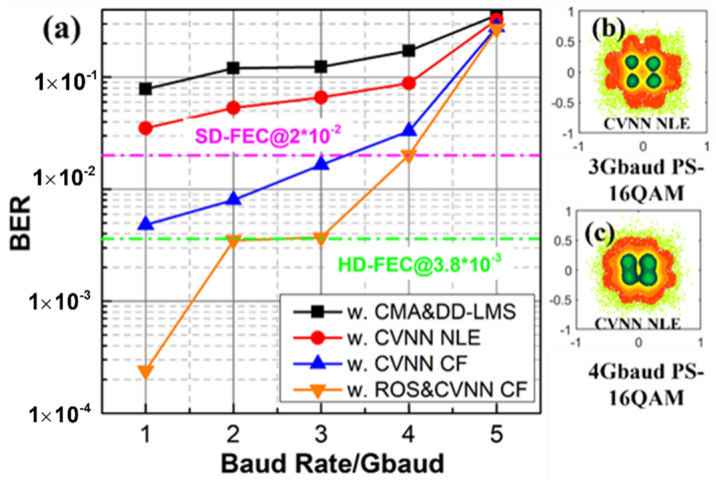
(**a**) The BER performance of PS-16QAM signals vs. the baud rate by using traditional DSP algorithms and nonlinear NN algorithms when the optical power into UTC-PD is fixed as 9 dBm. The constellation diagrams after CVNN NLE of (**b**) 3Gbaud and (**c**) 4Gbaud. Pink line: BER threshold for FEC coded symbols using soft decisions.

For the recovery of PS-16QAM, we also compare the BER performance of traditional DSP algorithms, CVNN nonlinear equalizer (NLE), CVNN classifier (CF), and our proposed CVNN CF combined with the ROS method in Figure 7. We can conclude that BER increases with the transmission speed. It is obvious that after traditional DSP algorithms, the constellation point recovery effect is not ideal with a high BER in Figure 7b,c, and there is only a little help for BER decreasing when employing the traditional CVNN equalizer with MSE loss function. Compared with CVNN NLE, CVNN CF exhibits better compensation for nonlinear distortion and generalization. Furthermore, BER of 4 Gbaud PS-16QAM is lower with the help of ROS combined with the CVNN CF, which is below the SD-FEC threshold of 2 × 10^−2^. Therefore, in our opinion, the best option is CVNN CE combined with ROS for 135 GHz PS-16QAM delivery over 4.6 km, although the complexity of CVNN NLE is lower.

**Figure 8:**
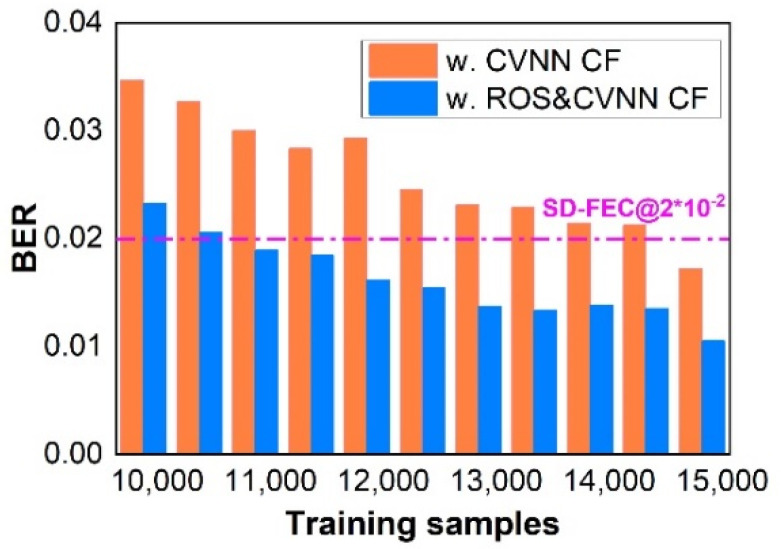
The corresponding BER vs. the training data size for 4-Gbaud PS-16QAM signals using CVNN classifiers and CVNN-ROS classifiers, respectively.

In Figure 8, BER varies with the size of the PS-16QAM training samples when the speed is 4 Gbaud. We can draw the conclusion that a longer training dataset is beneficial to BER reduction, but at the expense of efficient system capacity. As a result, a tradeoff exists between decision accuracy and efficient transfer speed. Furthermore, there is no doubt that the BER of PS-16QAM signals using the ROS combined with CVNN classifiers gives a better performance, and it can be reduced below 2 × 10^−2^ @ 20% SD-FEC when the length of the training set is 11,000, while CVNN CF requires 15,000 training samples. Therefore, it can be concluded that the CVNN-ROS classifier is also superior in terms of training sample reduction. 

**Figure 9:**
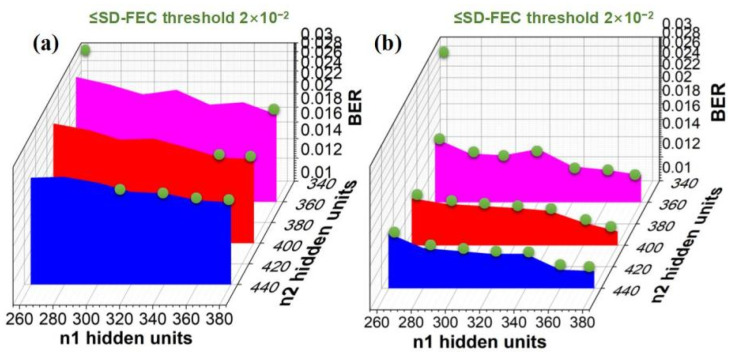
The BER performance of 4 Gbaud PS-16QAM signals with an optical input power of 9 dBm vs. the neuron cells *n*_1_ in hidden layer 1 and those *n*_2_ in hidden layer 2 by employing (**a**) CVNN classifier, (**b**) CVNN classifier combined with ROS.

As demonstrated previously, the complexity of the testing data is largely related to the NN structure. Therefore, with an optical power of 9 dBm, we further discuss the BER performance of 4 Gbaud PS-16QAM signal changing with the neuron units in two hidden layers of traditional CVNN and CVNN-ROS, respectively, in Figure 9. It is noted that green dots indicate when the BER reaches the Soft-Decision Forward Error Correction (SD-FEC) threshold of 2 × 10^−2^. Based on the calculation results, it can be concluded that increasing *n*_1_ or *n*_2_ can improve BER performance, though at the expense of complexity. Therefore, how to simplify the neural network architecture while ensuring decision accuracy by using complex training and ROS techniques is meaningful for the real communication system. The comparison results show that the amounts of cells *n*_1_ and *n*_2_ in hidden layers can be greatly decreased by utilizing the CVNN classifier coupled with ROS. Moreover, we select the minimum complexity point of these two neural networks among the green points, and compare their total number of multiplications in Table 1. The results show that smaller complex networks composed of a CVNN classifier and ROS outperform larger complex-valued networks. 

## 5. Conclusions

In this paper, 135 GHz fiber-wireless transmission using PS-16QAM over a 4.6 km wireless link is experimentally demonstrated. Compared with real-valued neural network (RVNN), the complex-value NN training method realizes phase information restoration and BER performance improvement. In particular, a CVNN classification algorithm coupled with ROS for a long-range D-band wireless transmission is very effective for handling both the learning imbalance and nonlinear noises of PS-16QAM. Thanks to the ML paradigms, we can achieve an 11.4-Gbit/s PS-16QAM mm-wave transmission at 135 GHz over 4.6 km. On the one hand, the CVNN-ROS classifier is superior in terms of training sample reduction. On the other hand, the smaller complex networks composed of the CVNN classifier and ROS also outperform larger complex-valued networks. Therefore, we believe that the combination of ROS data preprocessing and complex deep training techniques has an application prospect for the high speed PS-QAM wireless transmission. 

## Figures and Tables

**Table 1:** Network parameters of various CVNN schemes.

Type	Training Size	*n*_1_	*n*_2_	Minimum Multiplication	Complexity Reduction
CVNN	14,500	310	440	717,015	0%
CVNN-ROS	12,000	260	360	518,105	27.7%

## Data Availability

Not applicable.
